# Interface Block and Microstructure Evolution in Ultrasonic Welding of Aluminum

**DOI:** 10.3390/ma18122853

**Published:** 2025-06-17

**Authors:** Hang Qi, Fuxing Ye, Yingfan Wang, Kaiqi Sun

**Affiliations:** 1 Tianjin Key Laboratory of Advanced Joining Technology, School of Materials Science and Engineering, Tianjin University, Tianjin 300072, China; qihang0909@tju.edu.cn (H.Q.); 3018208222@tju.edu.cn (Y.W.); sunkq0413@tju.edu.cn (K.S.); 2 Key Lab of Advanced Ceramics and Machining Technology of Ministry of Education, Tianjin 300072, China

**Keywords:** ultrasonic welding, ultrasonic additive manufacturing, plastic flow, microstructure evolution, bonding mechanism

## Abstract

Ultrasonic welding, as a solid-state connection technology, has attracted considerable attention. The traditional ultrasonic welding sonotrode is not conducive to the study of the bonding mechanism of a straight interface, while the ultrasonic additive sonotrode does not have this problem. In this study, a special ultrasonic welding sonotrode was designed to form the joint, which is identical to ultrasonic additive manufacturing, to reveal its interfacial bonding mechanism between layers. Firstly, the linear metallurgical bonding density (LMD) of the joint is found to be positively correlated with welding time and negatively with welding pressure. Furthermore, the joint interface undergoes recrystallization after intense plastic deformation, with the obstruction of surface deformation by interface block resulting in the formation of a non-straight interface, which is beneficial to the formation of metallurgical bonding. Finally, a new concept of “Interface Block” was proposed, which can be applied to explain the formation of metallurgical bonding at the interface in ultrasonic additive manufacturing.

## 1. Introduction

Ultrasonic welding (UW) [1,2] is a low-temperature, solid-state bonding technology that is widely used for welding metal wires, foils, and plates. Ultrasonic additive manufacturing (UAM) [3,4] originated from ultrasonic welding and has been effectively utilized in the production of advanced composites such as laminated composites [5,6], metal–nonmetal composites [7,8], functional composites [9], and smart materials [10] (especially metal-embedded optical fibers [11,12]). Although these two technologies use different equipment, they have a very similar bonding mechanism and formation process for bonding surfaces. Due to its relatively low processing temperature, this technology is ideally suited for the preparation of metal matrix composites for embedded printed electronic circuitry [13,14] and integrated printed electronic conductors [15]. In the meantime, alloys comprising elements such as Fe [16,17], Al [18,19,20], Cu [21,22,23], Ni [24,25], and Zr [26] are employed in UW and UAM. An increasing number of novel material combinations are utilized in UW, rendering the technology highly promising. However, recent research on the interfacial bonding process and mechanism of materials has not advanced significantly compared to previous studies. The performance of composite materials is greatly influenced by the bonding mechanism between material interfaces. Therefore, it is necessary to conduct in-depth research on the bonding mechanism and bonding process.

Currently, most studies suggest that the bonding mode between interfaces in UW and UAM is metallurgical bonding. However, the specific bonding mechanism has not yet been determined. A number of studies have indicated that the oxide layer on the surface of a material is broken down due to friction, exposing the metal inside and contacting and diffusing it to form a bond at the atomic level. These studies explain the causes of material bonding, but do not investigate the bonding process. The objective of this paper is to investigate the interfacial bonding process, namely the plastic flow of the material and the evolution of the microstructure of the material at the interface, in order to gain a more in-depth understanding of the interfacial bonding mechanism.

**Figure 1:**
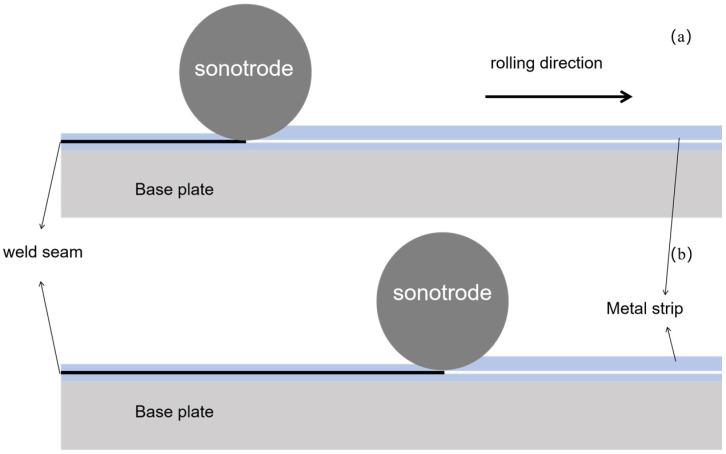
Schematic diagram of continuous motion process of UAM sonotrode: (**a**) initial position; (**b**) position after a period of time.

UAM is a continuous process. This is the most significant difference between UAM and UW. The sonotrode is rolled while the substrate is translated, creating a continuous weld zone between the materials, as shown in Figure 1. During the UAM process, the sonotrode moves across the substrate for a short duration (Δt) and a distance (Δd) close to zero. The sonotrode can be regarded as completing an ultrasonic spot welding process within Δt. Therefore, a continuous UAM process can be seen as a sequence of discontinuous ultrasonic spot welding processes. UAM and ultrasonic welding share the same principle, resulting in similar material interfaces produced by these two technologies. The interface of the weld joint in one of the ultrasonic spot welds was examined to determine the interface of the UAM material using the same parameters.

In addition, the sonotrode used by UW often features three-dimensional patterns that cause severe deformation of the material in contact with their edges because of stress concentration. This deformation causes the interfaces to mix and blend together, making them indistinguishable. In UW, interfaces that have not undergone severe deformation may still bond well, but the severely deformed interface masks it. In contrast, the acoustic surface used by UAM is relatively smooth with only slight roughness or shallow patterns, which do not cause severe deformation of the interface. Studying interfaces with mild deformation can help to explain the UW binding process and mechanism from the first principles. This is also the reason why a UW sonotrode similar to a UAM sonotrode has been designed in this study. Concurrently, the findings of this study offer a valuable contribution to the elucidation of the interface bonding mechanism in ultrasonic additive manufacturing.

In this paper, the instantaneous processing in the UAM process is simulated using a specially designed ultrasonic welding sonotrode. It is possible to produce joints with the same interface as ultrasonically reinforced composites by using specific processing parameters. A series of tests, including metallographic observation, are then carried out on the resulting joints. The purpose of these tests is to investigate the plastic flow of materials at the interface and the bonding mechanism of the materials.

## 2. Materials and Methods

In this paper, the original sonotrode was redesigned with the intention of utilizing an ultrasonic welding machine to achieve the same interface as that of UAM equipment.

**Figure 2:**
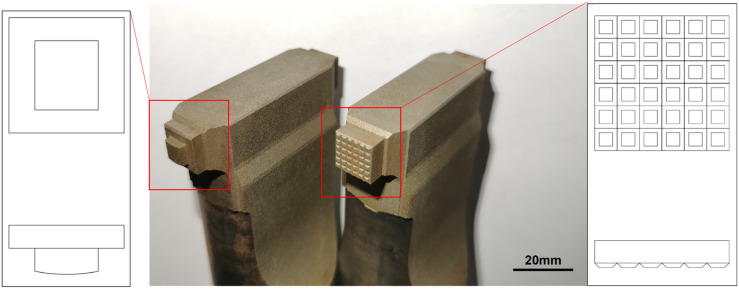
Comparison of two kinds of sonotrode: curved surface sonostrode (**left**) and conventional ultrasonic sonostrode (**right**) with their technical drawing.

The newly designed sonotrode is shown in Figure 2. The characteristic of the new sonotrode is that the contact surface with the workpiece is a smooth curved surface. The technical drawing is intended to represent the shape of the sonotrode, and the dimensions illustrated therein may deviate from the actual values.

In this study, the plastic flow and bonding process at the material interface of UW was investigated using AA 1060 strip with a thickness of 0.2 mm as the raw material and AA 1060 plate with a thickness of 1 mm as the substrate. The influence of each of the three parameters, namely welding pressure (3–4 bar), welding amplitude (20–30 μm), and welding time (0.03–0.08 s), on the material interface after welding is studied by controlling two of them unchanged and changing the other one. This range of parameters was selected based on previous research. The specific parameter settings are shown in Table 1. The cross-section of the joint perpendicular to the rolling direction (RD) was intercepted and metallographic specimens were prepared. These were then etched using Keller’s reagent (95% H_2_O, 2.5% HNO_3_, 1.5% HCl, 1% HF) and observed using an optical microscope (VERT A1, ZEISS, Oberkochen, Germany). The joint with a welding pressure of 4 bar, welding amplitude of 30 μm, and welding time of 0.05 s was selected for subsequent research. EBSD samples were prepared by polishing using a broad beam Ar ion milling and polishing system (Model 1061 SEM Mill, Fischione, Export, PA, USA) and analyzed by field emission scanning electron microscopy (JSM-7800F, JEOL, Tokyo, Japan).

## 3. Results and Discussion

### 3.1. Microstructure of the Interface

**Figure 3:**
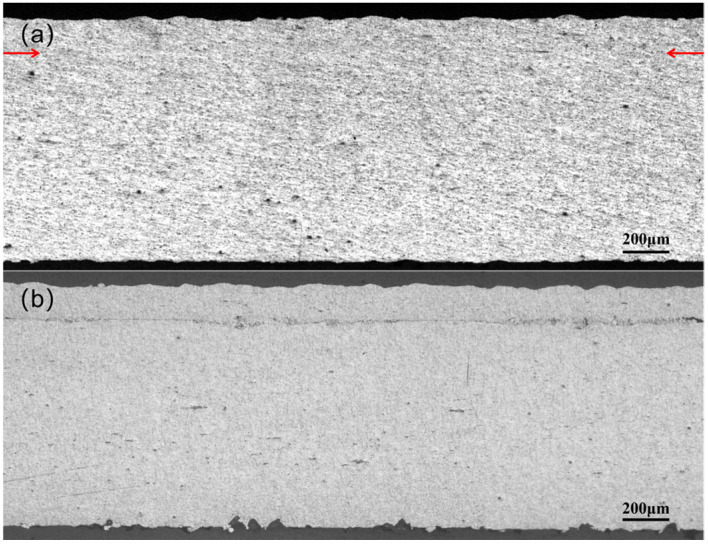
Microstructure of the RD surface of the joint: (**a**) unetched joint; (**b**) etched joint.

The microstructure of the joint cross-section was observed using an optical microscope, as shown in Figure 3. In Figure 3a, the RD surfaces of the unetched joints are completely welded, with a linear weld density of 100%. Furthermore, the interface between the upper and lower materials is indistinguishable from the interface position, which is marked with an arrow. In Figure 3b, the interface between the upper and lower materials can be observed following corrosion. The interface was originally the surface of the material, and a significant deformation occurred during the connection process, which elevated the interface’s energy level relative to the substrate. This rendered the interface more susceptible to corrosion, allowing for its observation after corrosion. The interface between the Al strip and the substrate is observed as a thin line that is roughly parallel to the upper surface of the strip. Furthermore, the thickness of the strip is uniform throughout. This indicates that the joint interface prepared by the special sonotrode used in this study is analogous to the joint interface prepared by UAM.

**Figure 4:**
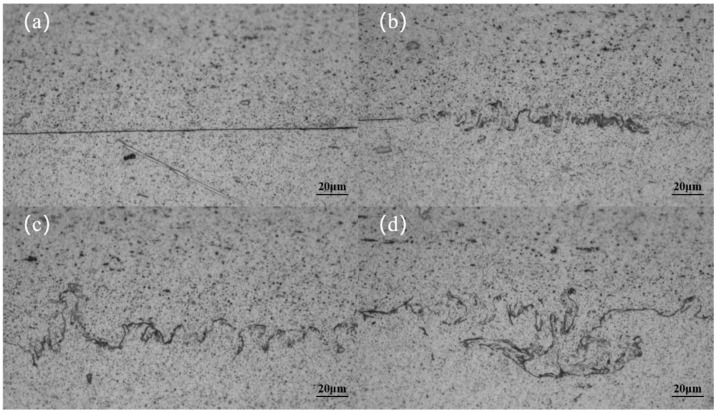
Microscopic structures of interfaces with different shapes: (**a**) linear, (**b**) wavy, (**c**) peak, and (**d**) vortex.

The microstructure of the interface was observed at higher magnification, revealing the existence of several typical shapes. These were subdivided into four types, namely linear, wavy, peak, and vortex, as shown in Figure 4. On both sides of the linear interface, there is no evidence of plastic deformation on the original surface of the material, and the flat surface of the original Al strip and plate is retained under lower magnification observation. In contrast, on both sides of the other three interfaces, the material undergoes significant plastic deformation, with the degree of deformation increasing in accordance with the wavy, peak, and vortex shapes, respectively. At some of the interfaces where the deformation was particularly severe, it was not possible to distinguish with certainty whether the material was from an Al strip or plate. In all of the joints obtained using the control variable method, these four distinct shapes of interfaces are observed. This indicates that their presence or absence is independent of the process parameters employed. It can be concluded that the wavy, peaked, and vortex interfaces represent essentially different forms of the same interface at varying degrees of deformation. The distinction between these three interfaces is solely based on the degree of deformation of the material at the interface. Consequently, they are collectively referred to as the non-straight interface, while the linear interface is referred to as the straight interface. During the evolution of the joint interface, a variety of morphologies have been observed. This indicates that the welding process of the material does not occur simultaneously throughout the interface. Rather, it can be postulated that the welding occurs first in some places, resulting in a final interface that is not identical in form everywhere.

**Figure 5:**
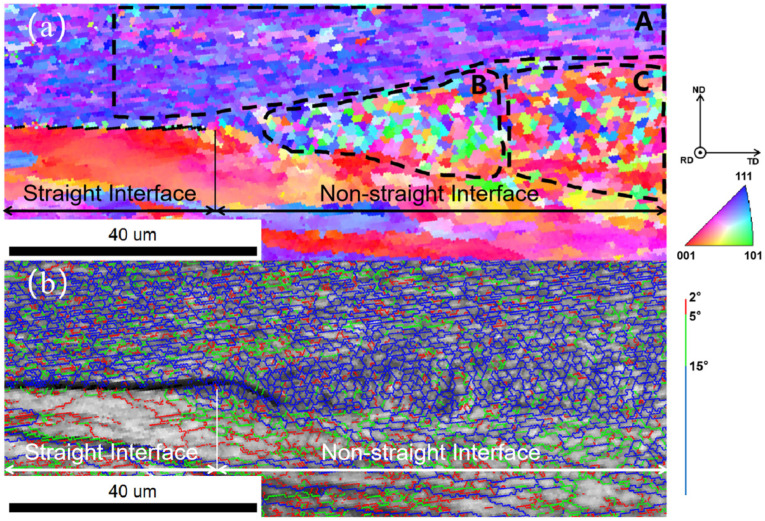
(**a**) IPF maps and (**b**) grain boundary maps at the junction of a straight interface and a non-straight interface.

In order to further investigate the difference between the straight interface and the non-straight interface, EBSD analysis was performed at the junction of these two interfaces, and the results are shown in Figure 5. At the straight interface, a clear demarcation is observed between the Al strip above and the Al plate below. Furthermore, the material near both sides of the interface is similar to the respective material away from the interface in terms of grain size, grain morphology, and grain orientation, which is typical of rolled grains. Given that the material at this location did not undergo significant changes at the grain level, it is likely that the straight interface was mechanical bonding. At the non-straight interface, the abrupt change in grain morphology between the Al strip above and the Al plate below is no longer evident. Instead, an area of a certain thickness at the interface exhibits a grain morphology that differs from that of the material on either side. The grains exhibit recrystallization, with no apparent preferential orientation of the grains. Additionally, the shape of the grains is not elongated, as is the case with rolled grains. Given that recrystallization of the material at this location has occurred and bonding between the materials has formed at the atomic level, it can be concluded that the bonding at the non-straight interface is metallurgical bonding.

**Figure 6:**
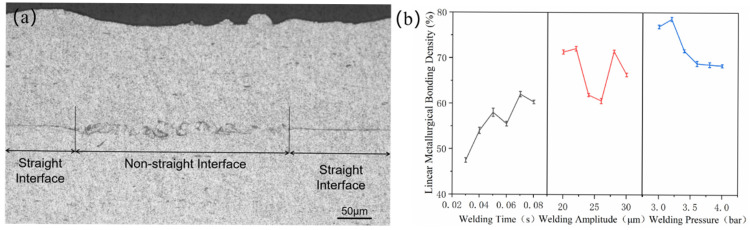
(**a**) Methods of delineation of straight and non-straight interfaces; (**b**) the influence of parameters on the LMD.

The non-straight interface is the metallurgical bonded interface, while the straight interface is the mechanical bonded interface, then the linear metallurgical bonding density (LMD) of the joint can be calculated according to

(1) $$\rho =\frac{L_{N}}{L_{N}+L_{S}}\times 100\%,$$

where *L*_N_ is the length sum of all non-straight interfaces in the weld and *L*_S_ is the length sum of all straight interfaces in the weld, and the results are shown in Figure 6. The LMD reflects the metallurgical bonding of the joint interface, which has an important influence on the performance of the joint interface. The LMD of the joint is positively correlated with the welding time and negatively correlated with the welding pressure, and there is no significant correlation with the welding amplitude. In the joints where the welding time varies, the LMD increases with the increase in the welding time, i.e., the non-straight interfaces increase while the straight interfaces decrease, and the phenomenon of the non-straight interfaces expanding and eliminating the straight interfaces with the increase in the welding time may exist. In the joints where the welding pressure varies, the welding pressure increases but the LMD decreases, probably because the higher welding pressure increases the friction between the material surfaces, limits the relative sliding and plastic deformation of the surface material, and the welding energy acts more on the deformation of the material as a whole rather than on the friction of the material surfaces. The reason for the lack of correlation of LMD in joints with varying weld amplitude may be due to the existence of an interplay between line weld amplitude and weld pressure, which has been reported in some of the previous literature [27]. As the welding pressure remains constant, a certain threshold is exceeded as the welding amplitude increases, resulting in a non-linear change in LMD.

**Figure 7:**
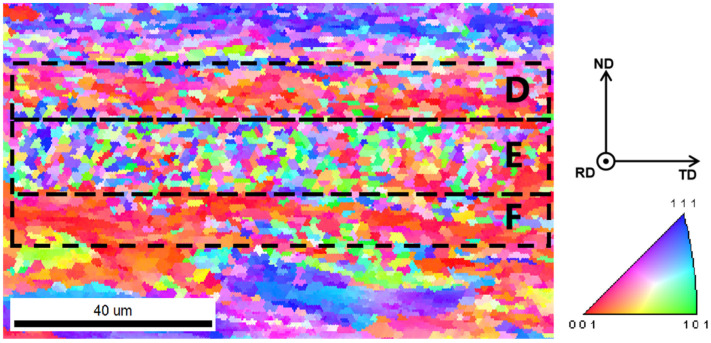
IPF maps of the central area of the non-straight interface.

The central area of the non-straight interface is examined in detail and the results of its EBSD analysis are shown in Figure 7. In the center of the non-straight interface, the interface has a certain thickness, which shows three areas with different characteristics from the materials on either side on the IPF maps. The grains in the central region are similar to recrystallized grains with a thickness of about 15 μm, with a smaller grain size than the materials on either side and no apparent elongation and selective orientation. There is also a layer between the central region and the materials on either side with a thickness of about 10 μm, whose grains have some degree of selective orientation but in a different direction from the materials on either side. During the evolution of the material interface, the grain morphology and microstructure of the material near the interface changed significantly after intense plastic deformation and temperature increase. In order to clarify the deformation and evolution process of the grains and interface materials in different areas of the interface, it is divided into six typical areas for study: area A is the upper Al strip with typical rolling texture; area B is the area of the non-straight interface closest to the straight interface; area C is the area in the non-straight interface leaning against area B; area D is the area away from the junction of two types of interfaces with the non-straight interface; area E and area F are the areas between area D and the materials on both sides.

The size distribution of the grains in the six areas was estimated and counted based on the IPF maps, and the results are shown in Table 2. While the average grain size of each area is relatively similar, the maximum grain size of the area in proximity to the interface is notably smaller than that of the substrate and the area in proximity to the substrate. Conversely, the standard deviation of the grain size at areas B, C, and E is observed to decrease. This indicates that the plastic deformation between the materials, as well as the recrystallization, result in a more uniform microstructure in these areas.

### 3.2. Microstructure Evolution of the Interface

**Figure 8:**
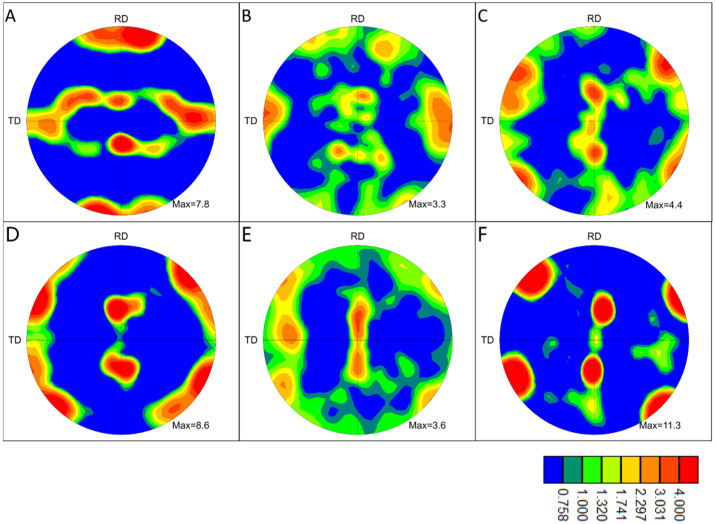
Pole figures of areas (**A**–**F**) that reflects the process of texture changes.

The {111} pole figures (subsequently designated as pole figures) of six distinct regions proximate to the interface were plotted, and the outcomes are presented in Figure 8. The pole figure of area A exhibits a typical body-centered cubic metal rolling texture, indicating that the microstructure of the material on the upper side does not change significantly before and after welding. The pole figure of area C exhibited a distinct change compared with area A, indicating the emergence of a novel texture. This suggests that the original rolling texture underwent a gradual dissolution during the vibration of the sonotrode and the plastic deformation of the material, accompanied by recrystallization. However, a new recrystallization texture, potentially a goss texture, emerged under the influence of the vibration of the sonotrode and the original texture. The pole figure in area B differs from those observed in areas A and C, indicating an intermediate state of texture change. This is likely due to the smaller deformation observed in this area, which has not yet resulted in sufficient recrystallization. In the central region of the non-planar interface, where recrystallization is more adequate, the pole figure of area E is similar to that of area C, reflecting an inadequate recrystallization texture. In contrast, areas D and F exhibit clear textures, likely due to the repeated plastic deformation of the first recrystallized grains under the influence of sonotrode vibration, which allows the development of recrystallization texture and the generation of obvious texture. During the dynamic evolution of the interface material, the intense plastic deformation of the interface material accumulates a large amount of stored energy of deformation, which, combined with the temperature increase caused by friction, facilitates recrystallization. The deformation of the material at the interface does not cease after recrystallization; rather, the repeated deformation along the direction of the sonotrode vibration causes the grains on both sides of the recrystallization area to develop into a new texture. The results indicate that the generation of recrystallization texture in areas C and E may not be solely attributable to the presence of the original texture but may also be influenced by repeated plastic deformation.

**Figure 9:**
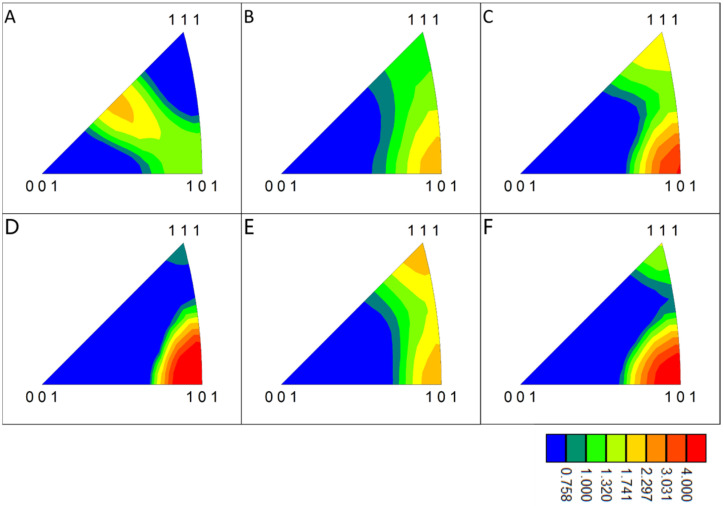
Inverse pole figures of areas (**A**–**F**) that reflects the process of texture orientation changes.

Figure 9 illustrates the inverse pole figures (IPFs) of the six areas. In areas D and F, the direction of the texture grains is parallel to the RD side. In contrast, in areas C and E, the orientation of grains is less concentrated, with a greater number of grains exhibiting <101> and <111> directions parallel to the RD. In area A, the orientation of the texture grains is parallel to the RD. Figure 9 provides a general indication of the deformation rotation process of grains during the welding process. The grains undergo a change in orientation according to the crystallographic features embodied from area A to area B, to areas C and E, and finally to areas D and F. Each area is named according to its characteristics during the evolution of the microstructure: area A is the original texture area; areas B and C are the initial recrystallization areas; area E is the recrystallization texture area; and areas D and F are the new texture areas. During the evolution of the microstructure, different areas appear successively and stop evolving at the end of the weld, remaining as the final observed microstructure.

### 3.3. Bonding Mechanism and Plastic Flow

**Figure 10:**
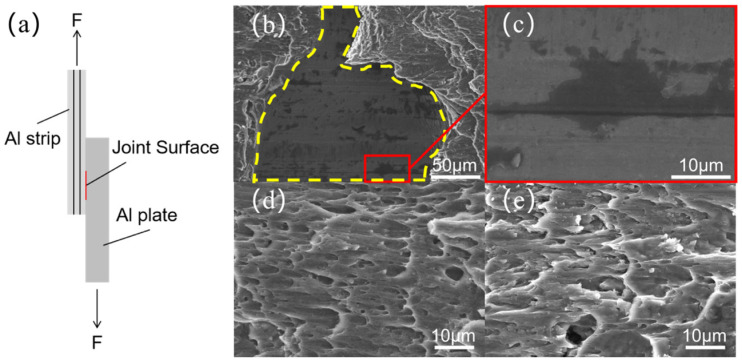
(**a**) Schematic diagram of a joint tensile test; fracture morphology of the joint: (**b**–**d**) Al strip; (**e**) Al plate.

Further research was conducted on the bonding mechanism of the joint interface, and tensile experiments were carried out on the joint in the direction indicated in Figure 10a. The good bonding of the joints is evidenced by the fact that the bonding strength of the joint surface exceeds that of the Al strip. It was observed that the majority of the joints broke at the Al strip, with a bonding strength exceeding 297.4 MPa. The fracture morphology of the Al strip side of the joint with low bonding strength was observed, as shown in Figure 10b,c. Figure 10b shows the fracture morphology of the straight interface, delineated by the yellow dashed line, and the fracture morphology of the non-straight interface, which occupies the remaining area. The fracture surface of the straight interface is observed to be flat. Figure 10c shows that the material surface at the straight interface did not undergo significant plastic deformation. Instead, the oxide film on the Al surface was solely ruptured and disappeared due to the effects of friction. The dark areas represent residual oxide films, while the light areas represent Al devoid of oxide films. The non-straight interface is a metallurgical bonding interface. Figure 10d,e shows the fracture morphology of the non-straight interface on the Al strip and plate, respectively. The images reveal the presence of dense ductile dimples, indicative of a ductile fracture mode. Consequently, the presence of non-straight interfaces serves to enhance the strength of the joint. More non-straight interfaces within the joint may enhance the mechanical properties of the joint as a whole.

**Figure 11:**
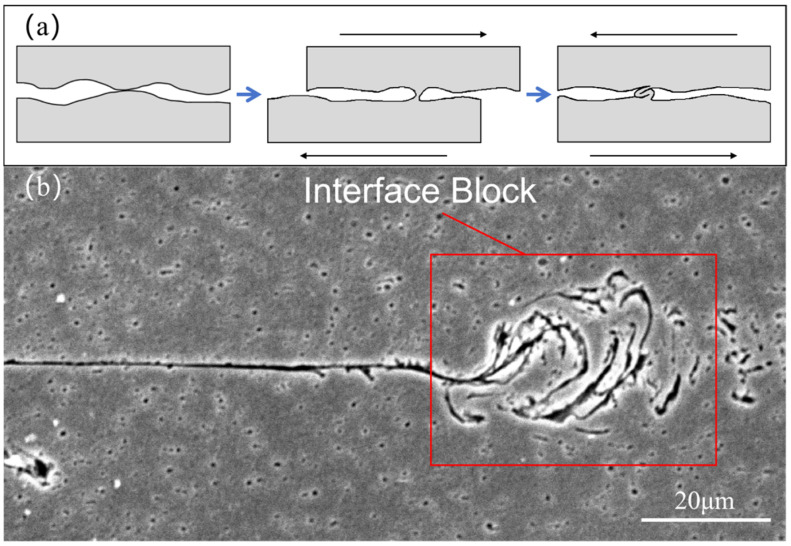
(**a**) Plastic flow mode of material interfaces; (**b**) microstructure of the interface block.

In a previous study on the plastic flow of materials during ultrasonic welding, a mode of plastic flow at the interface was proposed, as shown in Figure 11a [28]. The site where the interface first comes into contact and bonds is folded and extruded into a vortex-like structure during repeated motions. A comparable structure was identified in the present study and is designated as the “interface block,” as illustrated in Figure 11b. Due to the uneven surface of the original material, the surface micro-asperities take the lead in contacting and bonding [29,30]. Following the application of ultrasonic energy and sonotrode friction to the aforementioned locations, the formation of the interface block occurs as a result of the plastic flow process depicted in Figure 11a. The interface block has a higher degree of deformation and contains more material surfaces and dislocations with higher energy. This contributes to the recrystallization of the material. The location where the interface block forms serves as the origin of the non-linear interface. Following the plastic deformation of the material, the non-linear interface is extended and reaches a certain length, until the entire interface is fully welded.

**Figure 12:**
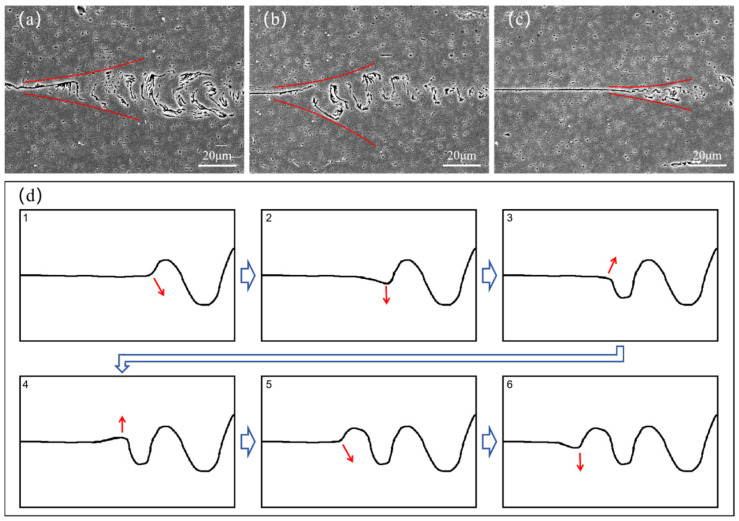
(**a**–**c**) Microstructure at the junction of straight and non-straight interfaces; (**d**) plastic flow mode of the interface.

At the junction of a straight interface and a non-straight interface, a sector-like transition area is often observed, as shown in Figure 12a–c. Based on the observation of a large number of microstructures and the study of the evolution process of the material grains at the junction of the two interfaces, a plastic flow mode that can form a non-straight interface is proposed, as shown in Figure 12d. Firstly, an interface block is formed between the upper and lower materials. Then, the surfaces of the upper and lower materials are driven by the sonotrode, with the horizontal motion blocked at the interface block, which can only carry out the deformation in the vertical direction (normal direction). After that, at the junction of the straight interface and the non-straight interface, following the frictional deformation, the interface arches in the upward or downward direction and grows upwards. When it reaches a certain height, the junction interface arches in the opposite direction, and this process is repeated. Finally, the aforementioned plastic flow process is repeated, with the non-straight interface expanding to the neighboring straight interfaces until the entire interface is transformed into the non-straight interface.

Combining the above results with the discussion, a mechanism of the interface block promoting the formation of metallurgical bonding at the interface is proposed. Firstly, prior to the formation of the interface, the micro-asperities on the surface first contact and combine. After the plastic flow process, the interface block is formed, which serves as the source point for the development of the non-straight interface. Secondly, the subsequent motion in the horizontal direction of the straight interface is blocked at the interface block, and then shifts to the movement in the vertical direction. Thirdly, following the plastic flow processes, a significant amount of plastic deformation is accumulated at the interface, with the proportion of non-straight interface continuing to increase. Fourthly, recrystallization occurs at the non-straight interface, with the surface of the original material disappearing, and a metallurgical bonded interface is formed. During the formation of the weld interface, the tissue at the interface undergoes recrystallization and forms a recrystallized texture, corresponding to area E in Figure 7. After that, the formed recrystallized grains are repeatedly deformed by the sonoteode, and the texture in the grains continues to develop, with a new layer of the texture formed between the recrystallized grains and the material on the two sides, corresponding to areas D and F in Figure 7. The areas next to the straight interface as well as in the non-straight interface with less plastic deformation and insufficient recrystallization correspond to areas B and C in Figure 5. The microstructure of the interface can be affected by welding time, welding amplitude, and welding pressure, thereby impacting the mechanical properties of the joint. However, the impact of these factors on the bonding process of the interface is unclear, and more in-depth research is necessary in the future.

## 4. Conclusions

This paper presents the results of an investigation into the use of a specially designed ultrasonic welding sonotrode to create joints with interfaces identical to those of UAM. The microstructure evolution of the interface and the interface bonding mechanism were also investigated. The main conclusions of this study are as follows:

1. The interface undergoes recrystallization after intense plastic deformation, resulting in a good metallurgical bonding. The LMD of the interface is found to be positively correlated with welding time and negatively correlated with welding pressure, while the effect of welding amplitude is complex.
2. The microstructure evolution of the weld interface was clarified. The material at the interface recrystallizes after intense plastic deformation and forms a recrystallization texture. After repeated deformation, the recrystallization texture develops into a new texture different from that in the original material. That is, it evolves in the order of original texture, recrystallization, recrystallized texture, and new texture.
3. A mechanism of an interface block promoting the formation of metallurgical bonding at the interface is proposed. The obstruction of surface deformation by the interface block results in the formation of a non-straight interface. Following a series of microstructural evolutions, a metallurgical bonded interface is formed.

## Figures and Tables

**Table 1:** Parameter setting of control variable method.

	Welding Pressure	Welding Amplitude	Welding Time
Group 1	3–4 bar	20 μm	0.05 s
Group 2	4 bar	20–30 μm	0.05 s
Group 3	4 bar	20 μm	0.03–0.08 s

**Table 2:** Grain size distribution in different areas.

	Average Grain Size (μm)	Maximum Grain Size (μm)	Standard Deviation (μm)
Area A	1.38	8.61	0.66
Area B	1.22	2.44	0.35
Area C	1.22	2.59	0.39
Area D	1.37	5.24	0.65
Area E	1.30	3.00	0.45
Area F	1.49	6.57	0.89

## Data Availability

The original contributions presented in the study are included in the article. Further inquiries can be directed to the corresponding author.
